# The Atlas of Chinese World Wide Web Ecosystem Shaped by the Collective Attention Flows

**DOI:** 10.1371/journal.pone.0165240

**Published:** 2016-11-03

**Authors:** Xiaodan Lou, Yong Li, Weiwei Gu, Jiang Zhang

**Affiliations:** 1 School of Systems Science, Beijing Normal University, Beijing, China; 2 College of Computer Science and Engineering, Northwest Normal University, LanZhou, China; East China University of Science and Technology, CHINA

## Abstract

The web can be regarded as an ecosystem of digital resources connected and shaped by collective successive behaviors of users. Knowing how people allocate limited attention on different resources is of great importance. To answer this, we embed the most popular Chinese web sites into a high dimensional Euclidean space based on the open flow network model of a large number of Chinese users’ collective attention flows, which both considers the connection topology of hyperlinks between the sites and the collective behaviors of the users. With these tools, we rank the web sites and compare their centralities based on flow distances with other metrics. We also study the patterns of attention flow allocation, and find that a large number of web sites concentrate on the central area of the embedding space, and only a small fraction of web sites disperse in the periphery. The entire embedding space can be separated into 3 regions(core, interim, and periphery). The sites in the core (1%) occupy a majority of the attention flows (40%), and the sites (34%) in the interim attract 40%, whereas other sites (65%) only take 20% flows. What’s more, we clustered the web sites into 4 groups according to their positions in the space, and found that similar web sites in contents and topics are grouped together. In short, by incorporating the open flow network model, we can clearly see how collective attention allocates and flows on different web sites, and how web sites connected each other.

## Introduction

The excess of information makes us no longer read-but skim and it’s becoming increasingly difficult to juggle all the news sources and keep on top of things, which brings us to the law of information [1], stated first by Simon, “wealth of information creates a scarcity of attention” [2]. So more and more scientists realize the attention crisis and try to find a better understanding of attention mechanism. However, due to the limitation of measurement and data collection, very few quantitative works are proposed until the coming era of big data. Especially the rapid development of social media provides us an unprecedented opportunity to know how people allocate their attention on information resources and what kind of behaviors are exhibited. Scientists started to study collective attention through news, movies, tweets and other social media to explore individual or social phenomena from a global scale [3–6]. Thus, collective attention becomes an expanding field and grows fast [7–9]. Conventional studies usually focus on information spreading on social networks. However, because bits can be copied freely, information flow breaks the flow conservativeness. Second, it is also hard to predict the popularity of an information resource because the connections on contents between different information pieces are ignored by this modeling approach. Therefore, people switch their focus of research on limited attention rather than unlimited information. These include the studies on how collective attention allocates [7], decays [8], and switches [9], and how information popularity [10, 11] and ranking methods [12, 13] affect.

Another line of collective attention study is along the dynamic aspect of attention [14–16]. Attention flow can be represented by a series of sequential actions of users such as clicks, posts, thumb ups and so on, which reflects the transitions of a large amount of users [17–19]. In computer science, a series of clicking behaviors of users is also called clickstream, which can be treated as a specific attention flow. With clickstream data, people can make predictions on users’ behaviors [20–22]. Nevertheless, most current works on clickstreams only focus on a single web site without thinking about the allocation of collective attention on different resources [23–26]. One of the reason is the lack of the methods for analyzing large scale data for attention flows but not merely the topological structures [27] and temporal evolutions [28]. Another reason is the scarcity of the data of collective attention flows on the entire web ecosystem level.

In this paper, we construct an attention flow network according to the sequential visiting data of a large number of users collected by China Information Center. We obtain the Atlas of Chinese web sites by embedding the entire network into a high dimensional space to obtain a geometric representation of collective attention flows, in which each web site is treated as an organism striving for user’s limited attention resembling energy flows in ecosystems. The embedding is based on flow distances between nodes. Flow distance is a novel quantity defined for attention flow networks which takes both network topology and collective behaviors of users’ consideration together [29]. In addition, we cluster the web sites based on the geometric representation and find that the sites with similar content are clustered into same groups. Finally, we study how attention flow, dissipation, web sites distribute on the space of embedding, and find that the distributions are very inhomogenous. All in all, this paper gives us a new perspective to see the attention flow patterns of Chinese people and the connection landscape of web sites shaped by collective attention clearly.

## Materials and Methods

### The data

The data is obtained from a Chinese Internet institution, which has collected more than 30000 online volunteers’ browsing data for about 5 years. To better analyze, we randomly sample the entire data set to build up a smaller data set containing 120 million records of all the clicking behaviors of 1000 users within one month. Each record containing the information of 64-bits time stamps, window names, the types of browses, URLs, and information of users, etc., is a switch of user’s jump between two URLs. First, we extract the domain names from the URLs since we only care about the inter-domain attention flows.

**Fig 1:**
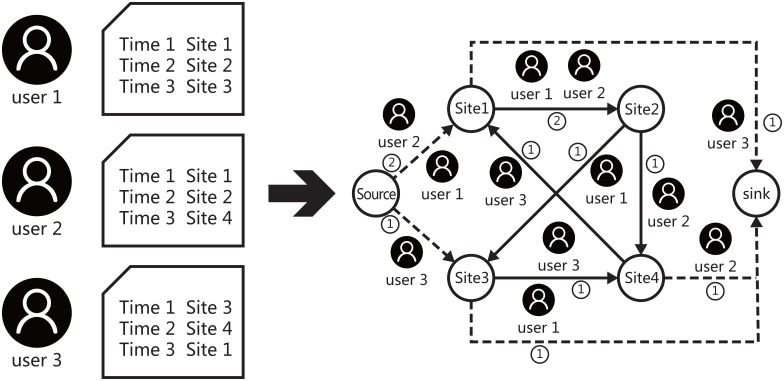
The construction of the attention flow network. In the left column, we list 3 users’ clickstream data which records the sites (URLs) and the visiting time ticks as examples. Accordingly, we can construct an open flow network model to depict the collective behaviors of these users. In the network, nodes are sites (URLs), and links are jumps between sites. The weight on each edge between *i* and *j* is the number of users who jump to *j* after his (her) visiting *i*. Notice that there are two special nodes: the source and the sink which represent the environment (offline world). If the time gap between any two records is longer than 30 minutes, we assume that this user jumps offline, which leading to a flow from the last visited site to the sink, and a flow from the source to the first site after 30 minutes.

We parse all the data to construct an open flow network, where a node is a site, the links denote the jumps between nodes and weights are flows. The whole process of network construction can be described as Fig 1.

Finally, we can get an (*N* + 2) * (*N* + 2) flux matrix denoted as *F*, where *N* is the total number of sites.

$$\begin{array}{c} F={\left\{f_{ij}\right\}}_{\left(N+2)(N+2\right)}, \end{array}$$ (1)

In which, node 0 and node *N* + 1 are the source and the sink, respectively. Thus we take the environment representing the offline world into account. Particularly, the existence of the flows from the source and to the sink is the unique merit of the open flow network compared with the conventional topological network and closed flow network [29]. Any entry in matrix *F* represents the number of users who visited *i* after immediate visit of *j* during a session. We assume that if the sequential visits of any two sites are within half an hour, all the visits are in one session. Otherwise, if the time lag is longer than 30 minutes between two URLs *i* and *j*, then we assume that the user jump offline from *i* during this time window and go back to visit *j* again. When this happens, we add an inflow from the source to the web site *j*, and an out flow to the sink from the site *i*.

### Major variables

To facilitate our discussion, we list several major variables and their calculation methods below.

1. Total attention flow.
  In our research, we define $\sum_{j=1}^{N+1}f_{ij}$ as the total outflow from node *i*, and $\sum_{j=0}^{N}f_{ji}$ as the total inflow to node *i*. It is interesting that the flow network that we have constructed in this way is balanced meaning that the inflow of each node balancing with the out flow. Thus, we define this value as total attention flow of node *i*, marked as *T*_*i*_(this quantity is also known as the traffic of web site *i*):
  $$\begin{array}{c} T_{i}=\sum_{j=0}^{N}f_{ji}=\sum_{j=0}^{N+1}f_{ij}, \end{array}$$ (2)
2. Dissipation flow.
  We call the flow from any node *i* to the sink as the dissipation flow. In the flux matrix, it is formed by the last column vector, namely:
  $$\begin{array}{c} D_{i}=f_{i,N+1}, \end{array}$$ (3)
3. Flow distances.
  The flow distance is defined as the average steps that one visitor jumping randomly from *i* to *j* for the first time along all possible flow paths. Since the conventional methods on network distances and random walks can’t be applied to the open flow network, we develop a new way to calculate the flow distance [29] according to the Markov transition matrix *M* with:
  $$\begin{array}{c} m_{ij}=\frac{f_{ij}}{\sum_{k=1}^{N+1}f_{ik}}, \end{array}$$ (4)
  Where, *m*_*ij*_ is the probability of one user jumping from *i* to *j* after his (her) immediate visit of *i*. Thus the flow distance of two web sites can be calculated as [29]:
  $$\begin{array}{c} l_{ij}=\frac{{\left(MU^{2}\right)}_{ij}}{{\left(U\right)}_{ij}}-\frac{{\left(MU^{2}\right)}_{jj}}{{\left(U\right)}_{jj}}. \end{array}$$ (5)
  Here, *U* = *I* + *M* + *M*^2^ + ⋯ = (*I* − *M*)^−1^ and (*U*)_*ij*_ is the pseudo-probability from *i* to *j* along all possible paths and I is the identity matrix with *N* + 2 nodes.
4. Symmetric flow distances.
  We can get a flow distance matrix *L* after calculating each pair of nodes’ distance, however, the distance from *i* to *j* is not the same as that from *j* to *i*. In order to embed the network into the Euclidean space, we need a symmetric distance. Therefore we define *C*, the symmetric flow distance as:
  $$\begin{array}{c} c_{ij}=l_{ij}+l_{ji}, \end{array}$$ (6)
  which can be explained as the average path length for a visitor going from *i* to *j* and back to *i*.

### Network embedding algorithm

We hope to give an atlas to depict the ecosystem of all Chinese web sites shaped by the collective attention flows. Therefore, we embed the entire network into a Euclidean space according to all the flow distances. The embedding is fulfilled such that all the Euclidean distances between any sites are closed to the symmetric flow distances as possible as it can. We use the spring algorithm [30] to do that. We suppose that there is a spring between two nodes and its natural length equals the symmetric flow distance of this nodes pair. Thus, they will be stretched or compressed unless all the springs are in natural lengths and the energy of all springs is minimized. Where, the energy is defined as the differences between the Euclidean distance and the symmetric flow distance for all node pairs. To realize the minimum energy state, we implement the following steps.

1. Initialization: we start by giving each node a random initial coordinate under a *D* dimensional space, where *D* is a free parameter.
2. Adjustment: through the spring algorithm, we calculate Euclidean distance between nodes and make errors as small as possible with the corresponding flow distance.
  $$\begin{array}{c} e_{ij}=\left|∥i^{D}-j^{D}∥-c_{ij}\right|, \end{array}$$ (7)
  Where, *i*^*D*^ is the position of node *i* in *D*−dimensional space. ∥*i^D^* − *j^D^*∥ is the Euclidean distance under *D* dimension and *e*_*ij*_ is the error. If the distance between two nodes is larger than the corresponding flow distance, the spring will exerts a pulling force to the nodes. Otherwise, there will be a repulsive force. The magnitude of the force is proportional to *e*_*ij*_ and this step will repeat until the total error is under a given threshold.
3. Fine tuning: we also implement the same spring algorithm as the previous step, however, the energy function is replaced by distortion defined as follow:
  $$\begin{array}{c} d_{ij}=max\left(\frac{∥i^{D}-j^{D}∥}{c_{ij}}-1,\frac{c_{ij}}{∥i^{D}-j^{D}∥}-1\right), \end{array}$$ (8)
  This metric is more sensitive than the error defined in Eq 7 because a small difference between Euclidean distance and flow distance will cause a large distortion. We can use the average value $\bar{d}$ to measure the overall performance of our embedding method:
  $$\begin{array}{c} \bar{d}=\frac{\left(\sum_{i=1}^{N}\sum_{i=1}^{N}d_{ij}\right)}{N^{2}}, \end{array}$$ (9)

## Results

### Flow distance (*l*_*ij*_) distribution

**Fig 2:**
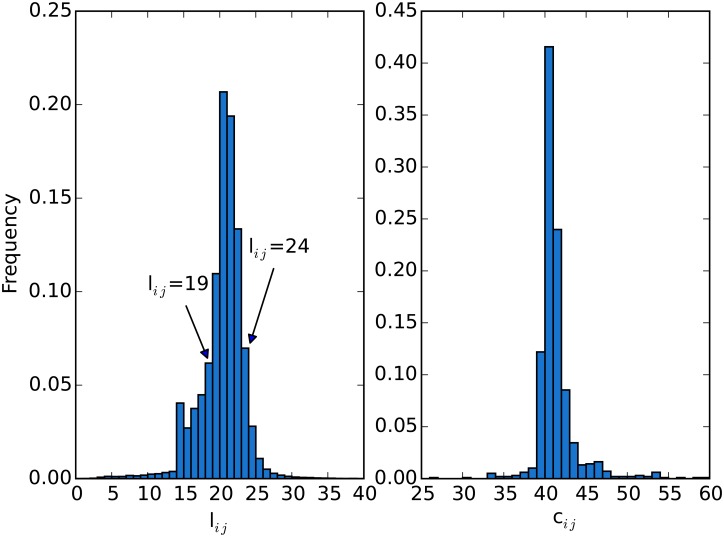
The distributions of flow distance (*l*_*ij*_) and symmetric distance (*c*_*ij*_).

We calculate the flow distance and the symmetric ones of all node pairs for 20737 web sites. And the distribution of the distances is shown in Fig 2, in which the X-axis represents the flow distance, and the Y-axis is the frequency of node-pairs. We can notice that the distribution has a shape with a high peak. In all those web sites, most of *l*_*ij*_s have flow distances between 19 and 24, especially, >40% are larger than 20 or 21. Only a few web sites have very small or large flow distances. That means for any two nodes, 20 steps of jump are needed in average for a user wandering on the WWW. That indicates the existence of a strong locality of the web surfing behavior for Chinese users.

### Ranking web sites according to the flow distances

We can define a new metric to measure the centrality of a web site. It is:

$$\begin{array}{c} \bar{c_{i}}=\sum_{j}c_{ij}, \end{array}$$ (10)

We find that famous web sites always have smaller average flow distances (*c*_*ij*_) than others because they have more traveling paths found by users to all other sites. Therefore, the less the average flow distance, the more central position a web site has. We can rank the web sites accordingly, and compare with other methods like clicks ratio and PageRank [31, 32], and the metric of total attention flow of each node. The ranking results for the top 17 web sites are listed in Table 1 in which the web sites are ranked by flow distances $\bar{c_{i}}$, and the number in bracket is the order of a site sorted by the corresponding method. We find that the rank by flow distance is quite different from the total attention flows, especially for the sites like tmall.com, 163.com, alipay.com and google.com, which have heavy traffic, but lower ranks in flow distances. It indicates that the high traffic web sites do not necessarily being more central than others. Therefore, traffics can’t determine the position of a site. Instead, baidu.com and qq.com are more central because of their short flow distances. This result also accords with our intuition, because baidu.com and qq.com are famous search engine and portal in China, respectively. They are the bridges between the real and the virtual worlds, leading much easier way to enter the Internet. When we compare with PageRank method which merely considers the topology of the network but not weights, we find that the outcome is quite similar. Thus, the ranking based on flow distance is more influenced by the network structure because flow distance not only considers the total traffics of the sites, but also the whole network’s topology, which makes it more objective to represent the importance of web sites.

**Table 1:** Ranking top 17 web sites according to flow distances and comparisons with other ranking methods.

rank	web name	flow distance	PageRank	Total attention flow
1	*baidu*.*com*	26.332(1)	0.0221(1)	105560(1)
2	*qq*.*com*	30.087(2)	0.0189(2)	57209(2)
3	*sogou*.*com*	33.035(3)	0.0138(3)	25979(4)
4	*taobao*.*com*	33.272(4)	0.0131(4)	35311(3)
5	*hao*123.*com*	33.626(5)	0.0120(6)	23295(5)
6	*sina*.*com*	33.818(6)	0.0122(5)	21711(7)
7	*weibo*.*com*	34.054(7)	0.0098(9)	21815(6)
8	163.*com*	34.979(8)	0.0108(7)	13890(12)
9	*sohu*.*com*	35.015(9)	0.0103(8)	15512(8)
10	360.*cn*	35.706(10)	0.0095(10)	14744(9)
11	*youku*.*com*	36.268(11)	0.0070(13)	14254(11)
12	*renren*.*com*	36.383(12)	0.0062(17)	11647(13)
13	*soso*.*com*	36.952(13)	0.0071(12)	8589(14)
14	*ifeng*.*com*	37.186(14)	0.0066(14)	7487(16)
15	*google*.*com*	37.202(15)	0.0077(11)	5938(17)
16	*tmall*.*com*	37.212(16)	0.0063(16)	14385(10)
17	*alipay*.*com*	38.252(17)	0.0057(15)	7723(15)

The numbers within the parentheses are the ranking orders according to the focus indicators.

### The atlas of the Chinese World Wide Web ecosystem

**Fig 3:**
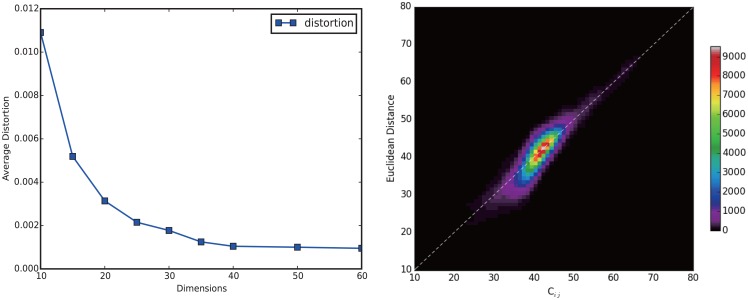
The effectiveness of the embedding. (A) The average distortion of embedding algorithm decrease with the embedding dimension. (B) The relationship between all the nodes’ Euclidean distances and the flow distances.

To visualize the atlas of the ecosystem of Chinese World Wide Web shaped by collective attention flows of users, we select a strongly connected sub-network containing the top 1000 web sites according to their traffics. And we try to embed them into Euclidean spaces in different dimensions by using spring algorithm, through which each site can obtain a coordinate. We test the metric of distortion changing with *D* (see Fig 3) and decide to select *D* = 20 as our final selection because it is the“elbow” of the curve meaning that the errors hardly drop by increasing dimension *D* after this point. In Fig 3(A), *X*−*axis* stands for dimension, and *Y*−*axis* represents average distortion. Fig 3(B) tests the effectiveness of our method by plotting the flow distance *c*_*ij*_ and the Euclidean distance between any nodes *i* and *j*, and the results clearly show that they are almost the same.

**Fig 4:**
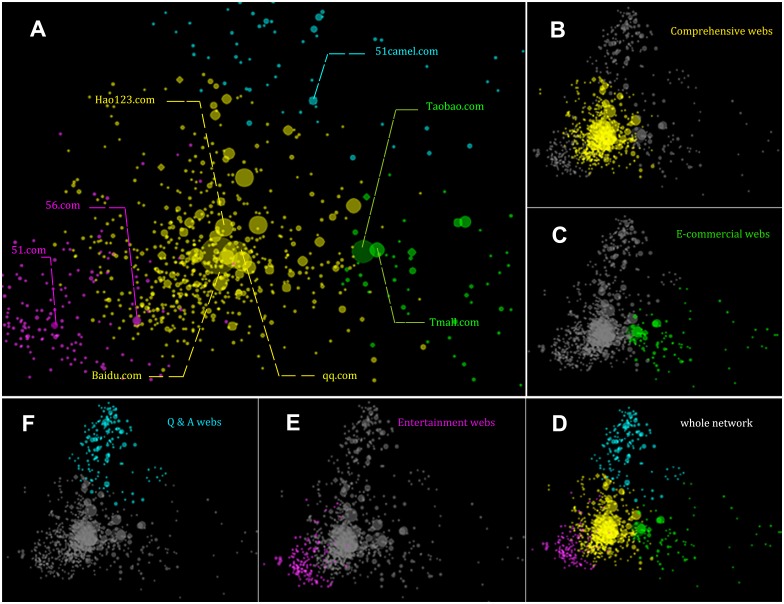
The visualization of embedding and the cluster analysis of the network. (A) is the amplification of central part of (D), the whole network. Node colors represent the categories of the web sites and the node sizes are proportional to the attention flows of the focus web sites. The other small figures are the same representations for different clusters, Comprehensive webs in (B), Shopping webs in (C), Entertainment webs in (E) and Q&A webs in (F).

To visualize, we use the PCA(Principal Component Analysis) method [33, 34] to project the embedding into a two dimensional space as shown in Fig 4, this is what we call the atlas of Chinese WWW ecosystem. In Fig 4, each node represents a web site, and their sizes are proportional to their total attention flows. It’s interesting to observe the asymmetry of the embedding. It seems the distribution of web sites is of high heterogeneity because several large clusters are obvious.

Therefore, we do the clustering analysis to see if similar web sites form groups. We cluster the web sites into 4 classes by the method of k-means algorithm according to the positions of all web sites in 20 dimensional space. Fig 4(D) shows the entire picture of the project in 2 dimensional space of the embedding, in which all nodes are colored according to their classifications, and Fig 4(A) magnifies the central area of Fig 4(D) and highlights some representative web sites. The web sites in different cluster can be clearly seen from Fig 4(B), the Comprehensive webs located in the central area (like baidu.com, sohu.com, hao123.com), Fig 4(C), the e-commercial web sites and payment sites (like Taobao.com), Fig 4(E), the entertainment web sites or story sites, and Fig 4(F), the Q&A web sites where people can get money or scores from answering questions. It’s interesting to find that the shapes of these clusters more or less show the properties of the sites and the preferences of the users. The questionnaire sites aggregate together more tightly than other sites owing to the tight connections between each other. Some of the shopping sites have long flow distance from the center. That’s because they are relatively isolated group shopping sites in different regions for local residences. This exposes users’ habit on the net that they always visit sites with similar contents sequentially.

What’s more, the positions of sites in the atlas represents their unique niches in the whole ecosystem. There is no doubt that baidu.com, the flagship of the search engine, is the center of the space, which means users may visit it frequently wherever they come from or go to, and other portals and engines all concentrate in central area too, such as hao123.com, sogou.com, and so on. It’s interesting to observe that taobao.com, the third largest traffic web site, is not as central as other web sites with smaller traffics. We suppose it is because Taobao is a special site just for online shopping representing a unique niche. Thus, this observation tells us that it is the flow distance but not the common traffics being the important hidden attribute for web sites.

In short, our clustering analysis reveals that the similar web sites are close each other in the space which validates our method of embedding based on flow distances. Second, the collective attention flows can reflect the similarity with contents between web sites and the ecological structure of Chinese WWW.

### The distributions of attention flow, dissipation, and web site

**Fig 5:**
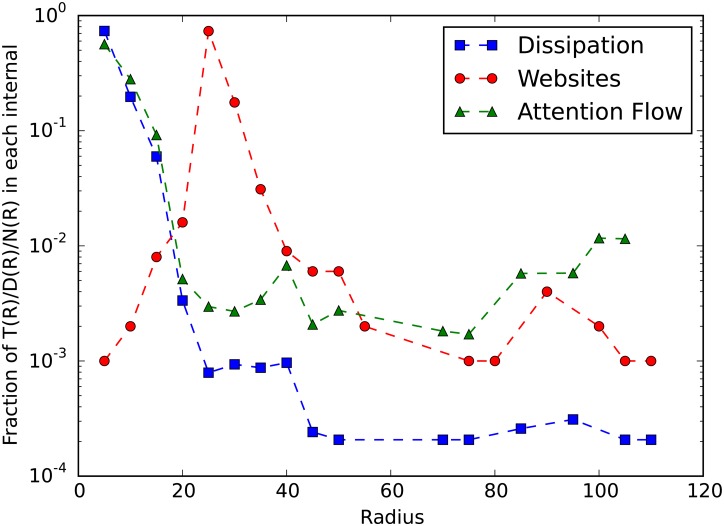
The distributions of attention flow, web site and dissipation along the distance from the center. *X*−*axis* represents the distance from the center, and *Y*−*axis* represents the percentage. The red, green, and blue curves are the numbers of web sites, total attention flows, and total attention dissipations.

To show how web site, total attention flow, and dissipation distribute in the D-dimensional space, we show the density curves and cumulative curves of these three variables along the distance from the center (baidu.com). Fig 5 shows that the distributions are extremely hierarchical because most sites are in the braid area with distances from the center 20 to 40, and only a few are close or far away from the center. However, it is interesting that not many attention flows concentrate on the same area, instead, they generally follow the principle of the farther the less (the green line). In another word, most attention flows concentrate in the areas close to the center where a few web sites locate but not the braid area where most web sites locate. In addition, we find that the dissipation distribution follows the similar way except that dissipation decays faster than attention flows along the distance from the center. These observations indicate that most Chinese users have a narrow interest spectrum meaning that they only visit large portal web sites like baidu.com and qq.com every day. But a few minorities who visiting very diversified web sites exist, they surf on the Internet with smaller jumping off probability because the dissipation rates are low for the web sites outside the central area.

**Fig 6:**
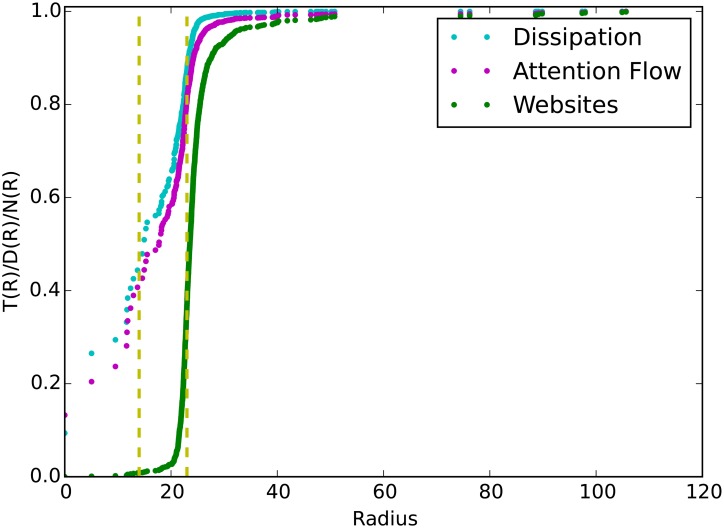
The cumulative distributions of attention flow, web site and dissipation. *X*−*axis* represents the distance from the center, and *Y*−*axis* is the cumulative percentages of the quantities of interest, the points represent the actual data and two yellow dashed lines are the quantiles.

The cumulative distributions of attention flow, dissipation and web site’ number along the radius further confirm the observation of the heterogeneities. From Fig 6, we divide the geometric space into 3 regions according to the quantiles of the attention flows (40% and 80%). In the first region, a ball around the center with radius approximately 13, there are nothing but 9 large web sites. We name this region as the core of the WWW in which 1% sites take over 40% of total attention flows. The second region called as interim, contains about 34% web sites and 43% attention flows along the radius from 14 to 23. It seems like 23 is the boundary, and people usually have the similar click pattern called reciprocation here when they surf on the net. What’s more, although many sites are in the third region, few of them are attractive. This may imply that the whole system is undergoing expansion right now.

## Conclusion and Discussion

In this paper, we exhibit the Chinese WWW ecosystem from a new perspective by using the collective flow data to better represent the connections between web sites. We construct an open flow network and embed it into a high dimensional Euclidean space based on flow distance, through which each site can obtain a unique position. Our method helps us to rank all the web sites according to the average flow distance from other sites, and leads to different results from the other methods. In the meantime, we further study the distributions of users’ attention flow and the dissipation, and find that all the web sites in the space can be separated into 3 regions. The portals and those sites with more attention flows locate in the central region, while they attract more than 43% attention flows. Only 34% web sites are in the second region and they only attract 40% attention flows. A large number of web sites concentrate in the third region, the peripheral part. However, they only have few attention flows because people seldom visit them. Thus, we can say that the Chinese WWW ecosystem is highly inhomogeneous. Finally, we have done a clustering analysis according to the positions in the embedding space of those web sites, and classify them into four clusters. It’s interesting to find that the sites with similar themes are closed each other and falling into same clusters. In general, although all the conclusions are drawn according to our data set, we believe that our method and basic conclusions can be generalized to other larger data sets. What’s more, the methodological innovation like flow distance, embedding method, can be applied to the open flow networks in other fields like trade networks, traffic flow networks.

It is worth and interesting to compare our work with Shi et al’s work on the English WWW world with the same method [17]. We find that although there are many commonalities between them, a few differences still exist. First, the shape of the embedding network in Shi et al’s study is more like a symmetrical ball and the distribution of all sites is homogeneous. On contrast, it is very asymmetrical and heterogeneous in our embedment. Our explanation is that the diversity of users’ visiting interests in our data set is not as large as the data set in Shi’s work, so the web sites are clustered into only a few blocks. Second, the centers of the whole embedment are different, they are Google.com and Baidu.com in English and Chinese worlds, respectively. However, these two centers are similar because they are all largest search engines in English and Chinese worlds. What’s more, Baidu.com has largest traffic in our data set but google.com does not. This indicates that our method can give us some heuristics on the niches (position) of each web site within the whole WWW ecosystem, and the niches are indifferent from traffics. Third, the shapes of the cumulative distribution curves along radical directions are different. The S-type characteristics of the cumulative distribution curves are apparent in Shi et al’s data set, but they are not in our data set because that there is large deviations in the heads of the S-curves which implying the monopolistic phenomenon on attention flows in Chinese WWW world.

We also left some interesting problems being worth of studying in the future. First, does there exist any other standards except flow distance for embedding the network into the space is worthwhile to be explored. And a way for validating the embedment is very necessary. Second, current study focuses on static properties of the snapshot of the whole WWW world, but the dynamical behaviors of all web sites and the entire ecosystem deserves for more attention and left for future studies. Third, one of the merits of our research is that it cannot only help us to see direct interactions between nodes, but also enable us to analyze the indirect connections between each node pair more clearly. Thus, our method is beyond the conventional traffic analysis which mainly focuses on direct contacts and interactions between web sites. Therefore, it should be able to evaluate each web site in a more comprehensive way. The potential applications include but not limit to predicting the amalgamation of web sites and the emergence of dark horse, as well as the recommendation for more appropriate online advertisement delivery.
